# Transforming Linear to Circular Polarization on Horn Antennas by Using Multiple-Layer Frequency Selective Surfaces

**DOI:** 10.3390/s22207838

**Published:** 2022-10-15

**Authors:** Adelaida Heiman, Razvan D. Tamas

**Affiliations:** 1 Department of Electronics and Telecommunications, Constanta Maritime University, 900663 Constanta, Romania; 2 Doctoral School of Electronics, Telecommunications and Information Technology, University Politehnica of Bucharest, 061071 Bucharest, Romania

**Keywords:** antenna radiation measurements, circular polarization, frequency-selective surface, multiple layers, pyramidal horn antenna, space communications, Yagi-Uda antenna

## Abstract

This article presents a technique for transforming the polarization of a pyramidal horn antenna by adding multiple layers of frequency-selective surfaces in front of the aperture, in order to rotate the direction of the electric field. Thus, two orthogonal components with the same magnitude, phase-shifted by 90°, are generated. Each frequency-selective surface consists of skewed *λ*/2 dipoles. Compared to other similar structures, our antenna system combines the field radiated from the horn aperture with the field scattered by parallel frequency-selective surface structures spaced on the same principle as that for designing a Yagi-Uda antenna array. The proposed horn antenna with multiple frequency-selective surfaces can be used as a feed element for a parabolic reflector antenna for maritime satellite communication systems in the X-band or in the lower part of the Ku band, or as part of a sensor for finding the direction of arrival of a wave, in order to orientate an antenna system. The concept was successfully validated on the basis of simulation and measurements. The proposed technique provides a close to unit axial ratio together with a 3 dB increase in gain compared to the conventional horn antenna, at low manufacturing costs.

## 1. Introduction

Antennas with circular polarization (CP) are needed in many modern communications systems, and many comprehensive studies have been conducted with the aim of producing CP waves for different applications [1,2,3,4,5].

Antennas for space communication systems must provide circular polarization, given that the polarization of radio waves rotates when crossing the atmosphere [6]. The horn antenna is one of the most widely used microwave antennas due to its simple structure, convenient feeding method, and low loss. Although cheap and highly directional, pyramidal horn antennas provide a linear polarization. Polarization transformation can be achieved by three main methods. The first method consists of placing two probes in the waveguide perpendicular to each other, one placed on the wide wall of the waveguide and the other one on the narrow wall. The signal is applied to the probes by means of a hybrid divider, yielding two signals of equal power, but out of phase by 90° [7]. The second method consists of placing a single probe in the waveguide at an angle of 45° with respect to the orientation of the horn antenna [8,9]. The third method consists of passing the linearly polarized signal through a layered polarization filter. The filter is inclined at an angle of 45°, so that one of the components of the electric field passes unchanged, while the other one passes with a certain delay [10,11].

The performance of a circularly polarized (CP) horn antenna mainly depends on the type of circular polarizer, which could be either a metallic septum polarizer [12] or a built-in dielectric plate circular polarizer [13]. These antennas have complex structures and a high cost.

A low-cost, general-purpose pyramidal horn antenna can be easily converted into a CP antenna if a linear-to-circular polarization converter is placed in front of the aperture.

Recently, frequency-selective surfaces (FSS) [14] or metasurface-based converters [15,16,17,18,19] have become an important research direction. There are many studies on horn antennas with phase compensation by FSS aperture loading in order to achieve a high gain and a narrow beam [20,21]. Single-layer FSS structures are usually used to filter the signal in a certain frequency band [22,23,24]. Conversely, multiple layers of FSS are used to change the polarization of the antenna and to increase its gain [11,25,26,27,28].

In [16], a polarization converter based on a multilayer non-resonant FSS was proposed. The size of the unit cell was much smaller than the wavelength. To generate enough phase difference between two electric field vectors with different directions, the FSS structure included a three-layer patch with truncated corners and a two-layer grid line, etched on the four dielectric slabs. This structure was efficient, but its complexity may have an impact on the manufacturing costs. As in [11,17], the polarization converter presented in [18] was placed at a certain distance away from horn aperture. Compared to the installation of a polarization converter inside the feeding waveguide, loading a polarization converter in the horn aperture [19] may effectively reduce the overall, vertical size of the antenna and mounting on the antenna is easier.

This article presents an alternative method for transforming antenna polarization, basically by adding multiple layers of frequency-selective surfaces in front of the pyramidal horn aperture, in order to rotate the direction of the electric field by 45° and consequently, to form two orthogonal components with the same magnitude and phase shifted by 90°. Adding an FSS multilayer structure also increases the gain of the antenna. As opposed to other similar approaches, frequency-selective surfaces are much simpler and consist of skewed, half-wave linear dipoles, since they provide a good response in polarization. Compared to other multiple-layer structures, the FSS layers were spaced so as to operate on the principle of a Yagi-Uda antenna array. As a result, the radio waves emerging from each element are transmitted with such delays that the individual fields add up constructively in the main direction of radiation, and destructively in the opposite direction. The horn aperture actually plays the role of the vibrators in a Yagi-Uda array. Circular polarization is eventually achieved by appropriately adding the transversal field components directly originating from the horn antenna, and from the passive elements, respectively.

To test our concept, ten FSS configurations were investigated on the basis of simulation, and the best four were fabricated and successfully validated by means of measurements. Our antenna was designed for applications operating in the X-band around 12 GHz, or in the lower Ku band, typically below 13 GHz. It can be used as a feed element for a parabolic reflector antenna for maritime satellite communication systems, for fixed Earth-to-satellite communications (the uplink is typically performed using the frequency band from 11.45 GHz to 11.7 GHz), or as part of a sensor for finding the direction of a wave in order to point an antenna system in that direction and therefore to ensure maximum power transfer.

This paper is organized as follows. Section 2 presents the method for obtaining a dual polarization by using passive radiators, Section 3 presents the proposed radiating system consisting of a pyramidal horn antenna with a layered FSS structure placed in front of the aperture, and Section 4 presents the conclusions.

## 2. Transforming Wave Polarization Using Arrays of Passive Radiators

### 2.1. Generating a Dual Polarization Using a Linear Radiator Tilted with Respect to the Direction of the Incident Electric Field

**Figure 1:**
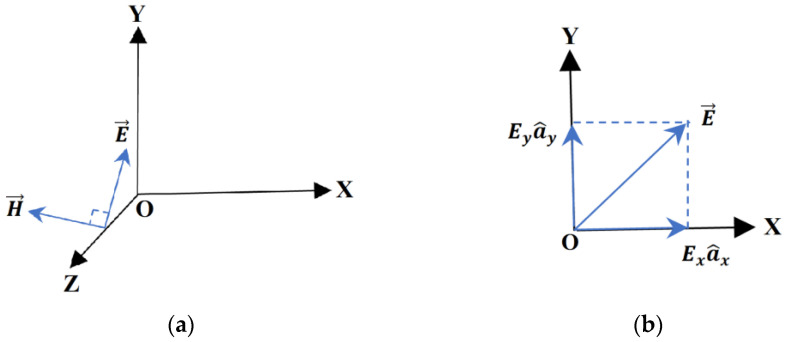
TEM wave propagating along the O*z* axis (**a**) and electric field strength components (**b**).

Consider a TEM wave propagating along the O*z* axis (Figure 1a). The electric field intensity vector can be decomposed along the axes O*x* and O*y*, as shown in Figure 1b, and can be written as:

(1) $$E=E_{x}\left(z,t\right){\hat{a}}_{x}+E_{y}\left(z,t\right){\hat{a}}_{y}$$

where

(2) $$E_{x}\left(z,t\right)=E_{x,0}\cos\left(\omega t-k_{0}z\right)$$

(3) $$E_{y}\left(z,t\right)=E_{y,0}\cos\left(\omega t-k_{0}z+\Delta \Phi \right)$$

(4) $$k_{0}=\frac{2\pi }{\lambda }$$

and ΔΦ is the initial phase shift between the two components of the electric field.

If $\Delta \Phi =\frac{\pi }{2}$ then:

(5) $$E=E_{x,0}{\hat{a}}_{x}\cos\left(\omega t-k_{0}z\right)+E_{y,0}{\hat{a}}_{y}\sin\left(\omega t-k_{0}z\right)$$

and if, in addition, *E_x_*_,0_ = *E_y_*_,0_ = *E*_0_ then

(6) $$E=E_{0}\left[{\hat{a}}_{x}\cos\left(\omega t-k_{0}z\right)+{\hat{a}}_{y}\sin\left(\omega t-k_{0}z\right)\;\right]$$

In that case, **E** = ct, and the polarization of the wave is circular.

**Figure 2:**
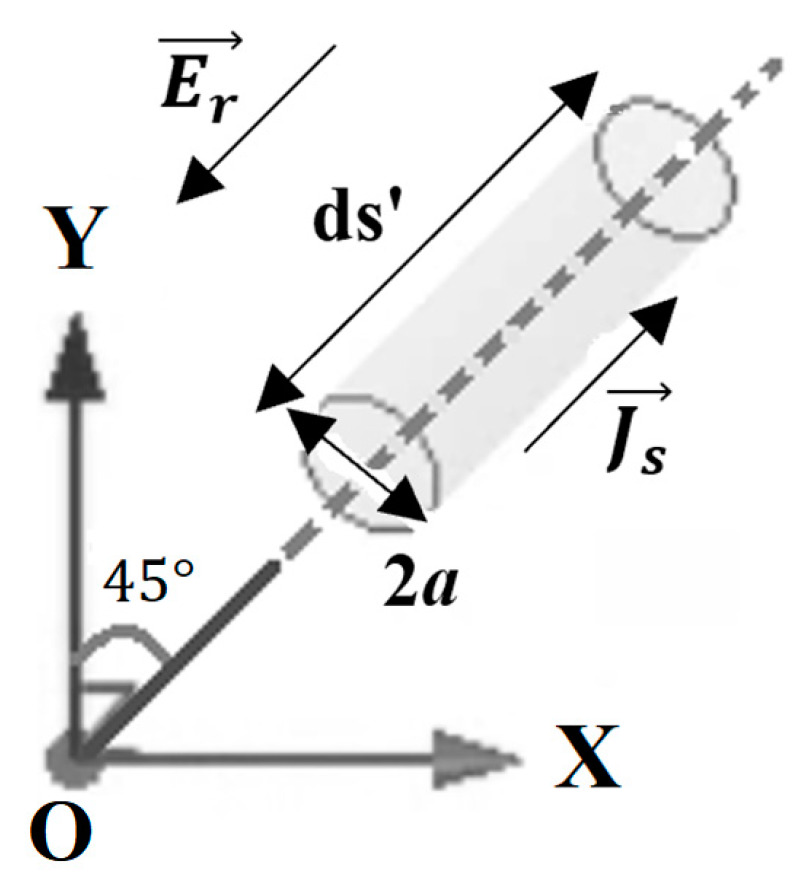
Linearly polarized wave incident to an elementary dipole.

Now let us consider a linearly polarized wave along the O*z* axis, incident on an elementary dipole located in the plane (*x*O*y*), inclined at 45° with respect to the O*y* axis (Figure 2). The radiator is considered to have a circular section of finite radius, a≪λ and an infinitesimal length ds’.

By applying the boundary conditions, the superficial current density induced on the surface of the radiator will be:

(7) $$J_{s}=\frac{2}{\sqrt{2}}\hat{n}\times H_{i}$$

and the induced current can be found by integrating the current density on the side surface of the radiator:

(8) $$I=2\pi a\frac{2H_{i}}{\sqrt{2}}=4\pi a\frac{E_{i}}{\eta \sqrt{2}}$$

where η is the free space wave impedance.

The reradiated electric field in a direction perpendicular to the current element will be:

(9) $$E_{r}=-jk_{0}\frac{E_{i}}{\sqrt{2}}\frac{a}{r}\exp\left(-jk_{0}r\right)\;ds^{\prime }$$

The vector $E_{r}$ decomposes into two orthogonal components, one along the O*x* axis and the other one along the O*y* axis, of magnitude

(10) $$E_{rx}=E_{ry}=-jk_{0}E_{i}\frac{a}{2r}\exp\left(-jk_{0}r\right)ds^{\prime }$$

**Figure 3:**
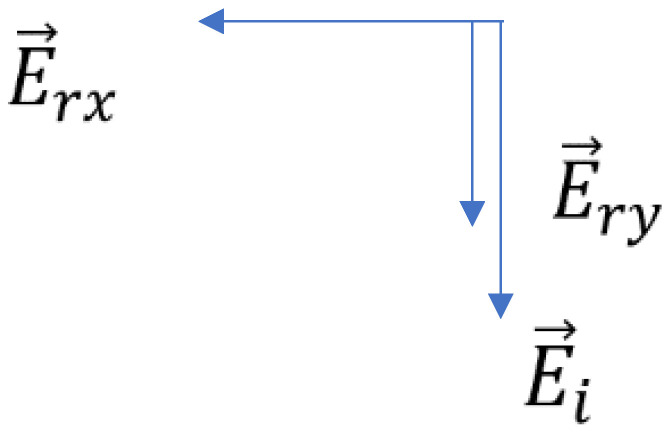
Components of the incident and reradiated electric field.

The components of the total electric field (incident and reradiated) are shown in Figure 3. Their magnitudes are

(11) $$E_{x,tot}=-E_{rx}=jk_{0}E_{i}\frac{a}{2r}\exp\left(-jk_{0}r\right)ds^{\prime }$$

(12) $$E_{y,tot}=E_{i}+E_{ry}=\frac{E_{i}}{2r}\left(2r_{0}-jk_{0}ds^{\prime }a\right)\exp\left(-jk_{0}r\right)$$

where $r_{0}$ is the distance between the primary radiation source, i.e., the horn aperture, and the elementary dipole.

When $r_{0}\gg a$, the first term in the expression of $E_{y,tot}$ is dominant and $E_{x,tot}\;$ and $E_{y,tot}\;$ will be out of phase with each other by approximately π/2.

The magnitude of the $E_{rx}$ component can be increased by successively exciting arrays of passive radiators, as is the case for Yagi-Uda antenna arrays. Conversely, such an array of passive radiators would not increase the magnitude of $E_{ry}$ to the same extent as for $E_{rx}$, given the magnitude and phase relationship between the terms in $E_{y,tot}$.

### 2.2. FSS Unit Cells Potentially Usable as Polarization Transformers

**Figure 4:**
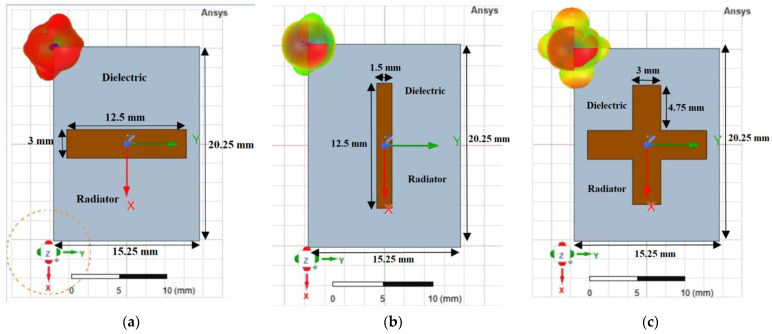
Unit cell: front and radiation pattern in *x*O*y* plane for radiators with “−”, “|” and “+” shapes.

Three types of unit cells (Figure 4a–c) made on a 1-mm-thick FR4 plate consisting of a single copper layer and a dielectric substrate were analyzed. The unit cells shown in Figure 4 consist of passive *λ*/2 dipoles resonating at 12 GHz. The study was carried out for horizontal, vertical, and cross radiators to observe whether a circular polarization could be obtained. The excitation was applied through a Floquet port. Simulations were performed for different widths of the radiators varying between 1.5 mm and 3 mm with a step of 0.5 mm. The frequency-selective structures were rotated at an angle of 45° with respect to the horn aperture axis. To use such a structure to generate a circular polarization, one component of the electric field should pass without the phase changing through the FSS, whereas the other component should be out of phase by 90°.

**Figure 5:**
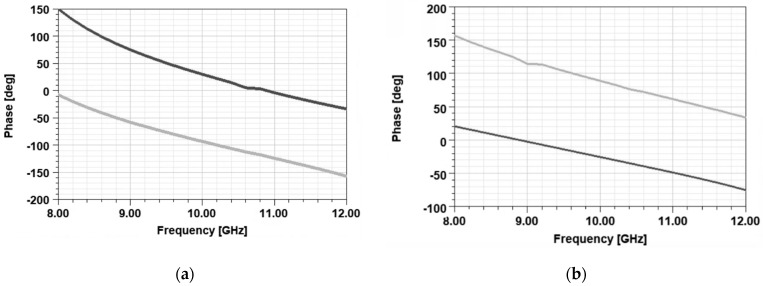

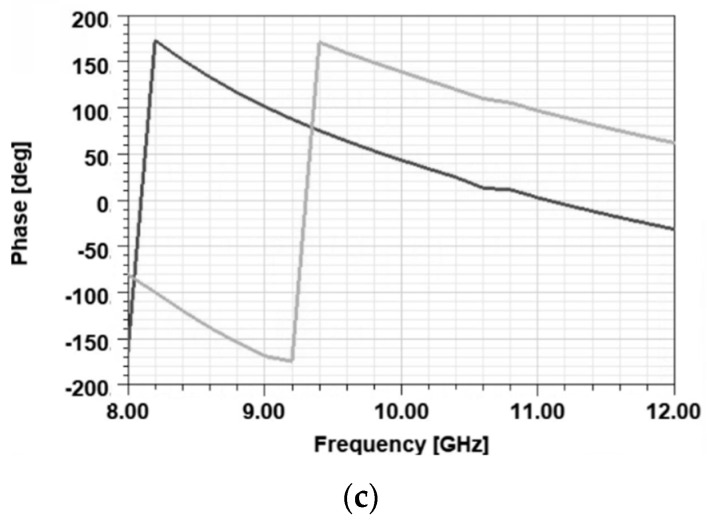
Phase of the two orthogonal electric field components as a function of frequency (grey line—*y* component; black line—*x* component).

Figure 5a–c shows the phase difference between the two orthogonal *E*-field components. It can be noted that a phase difference of 90±10° is obtained over the frequency band of interest.

## 3. Converting a Pyramidal Horn Antenna into a Circularly Polarized Antenna

### 3.1. Analysis of the Original Pyramidal Horn Antenna

**Figure 6:**
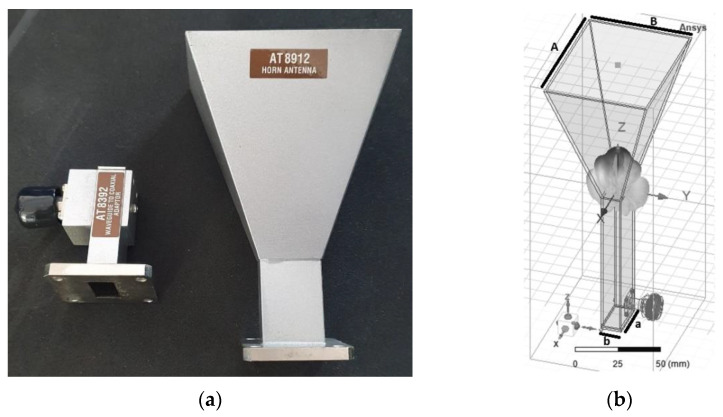
Pyramidal horn antenna: radiator and coaxial-to-waveguide adapter (**a**), and simulation model (**b**).

We departed from an existing, general-purpose horn antenna (Figure 6a) designed for the X band (8 ÷ 12 GHz). The transverse dimensions of the waveguide were a=22.86 mm and b=10.16 mm, and the length was 90 mm. The critical frequency of the fundamental mode was 6.55 GHz, and the critical frequency of the next higher mode is 13.11 GHz. The horn aperture length was *A* = 81 mm, the width *B* = 61 mm, and the height of the horn was 86 mm. The length of the monopole exciting the waveguide was 7.5 mm, with a radius of 0.76 mm, and its position relative to the short circuit wall was 11.5 mm.

**Figure 7:**
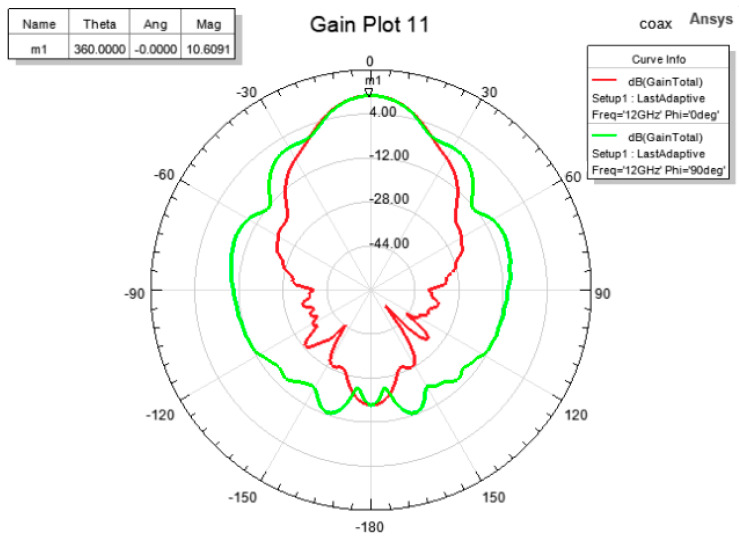
Simulation of the E-plane and H-plane radiation patterns of the horn antenna at 12 GHz.

**Figure 8:**
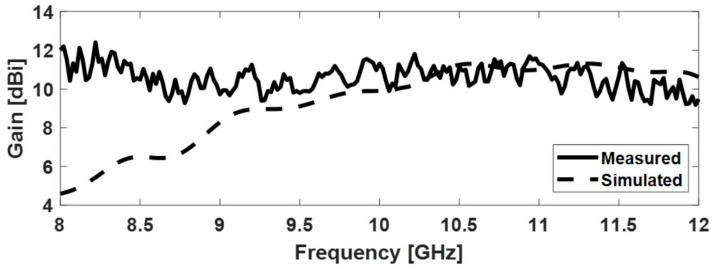
Gain of the pyramidal horn antenna in the main direction of radiation.

In simulations, a Perfect Electric Conductor (PEC) was considered as the material for the waveguide and the pyramidal horn, and copper for the monopole (Figure 6b). Figure 7 shows the radiation patterns in the E and H planes at 12 GHz, and Figure 8 displays the gain variation over the X-band, in the main direction of radiation. At 12 GHz, the simulated gain was 10.6 dBi, whereas the measured figure was 9.22 dBi; higher discrepancies can be noted at frequencies below 9 GHz, possibly due to errors occurring when measuring the physical size of the inner components of the coaxial-to-waveguide adapter. As further development including FSS polarization transformers was focused on frequencies around 12 GHz, no other optimization was performed for simulation at the lower frequencies in the X-band.

**Figure 9:**
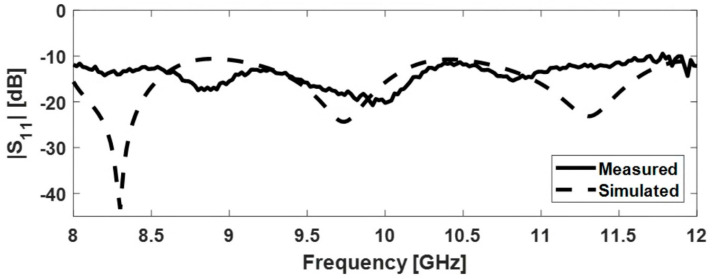
Input reflection coefficient for the pyramidal horn antenna.

As shown in Figure 9, the magnitude of the reflection coefficient is below −11 dB over the entire frequency band, both for measured and simulated figures.

### 3.2. Radiating Systems with Circular Polarization Consisting of a Horn Antenna and FSS Layers

The design of the frequency-selective surfaces went through two stages. The first stage consisted of determining the optimal number of elements of a single FSS layer; this study was carried out for cross-shaped radiators (+). The second stage aimed to establish the optimal number of FSS layers and to optimize the shape of the radiating elements.

#### 3.2.1. FSS Structure with N × N Cross-Shaped Elements

The system operates on the principle of a Yagi-Uda antenna system consisting of *λ*/2 dipole antennas [29]. The horn antenna actually replaces the vibrators and the reflector and the FSS elements act as directors.

**Figure 10:**
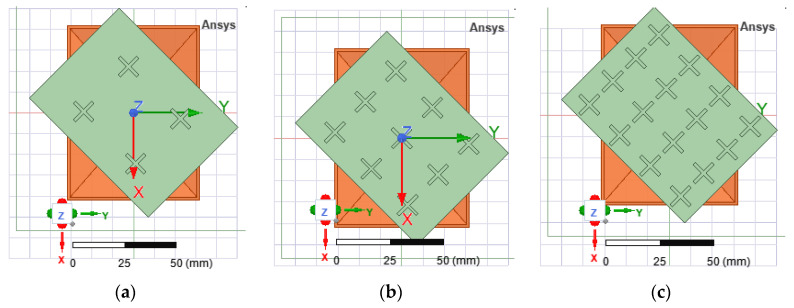

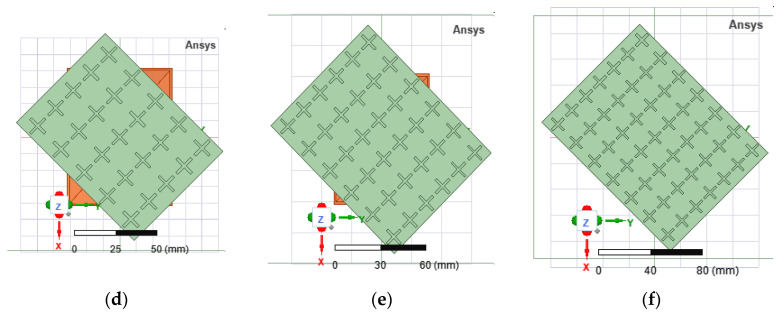
FSS structures with *N* × *N* elements in the aperture of the horn antenna.

An FSS structure with cross-type radiators (+), rotated at an angle of 45°, was added in front of the pyramidal horn aperture. The simulations were carried out for several elements of the FSS structure, *N × N* (Figure 10a–f): 2 × 2, 3 × 3, 4 × 4, 5 × 5, 6 × 6 and 7 × 7, respectively. The radiators have an electrical length of λ/2 at 12 GHz, that is, 12.5 mm, and a width of 1.5 mm. The FSS structures with 2 × 2, 3 × 3 and 4 × 4 elements have the same size as the pyramidal horn aperture, i.e., 81 mm × 61 mm; the distance between elements implicitly decreases with increasing *N*. For the FSS structures with 5 × 5, 6 × 6 and 7 × 7 elements, the gap between radiators is kept the same as for the structure with 4 × 4 elements, and therefore, the FSS size for these structures changes as follows: 100 mm × 76.25 mm for 5 × 5 elements, 116 mm × 91 mm for 6 × 6 elements, and 140 mm × 106.75 mm for 7 × 7 elements. In this study, the distance between the pyramidal horn aperture and the FSS structure was set to zero. The resulting figures of merit for the six types of structures are given in Table 1.

Following the performance analysis of the radiating system consisting of the horn antenna and different types of FSS structures, described above, it turned out that the best option would be an FSS structure with 4 × 4 radiators.

#### 3.2.2. Multiple-Layer FSS Structures

**Figure 11:**
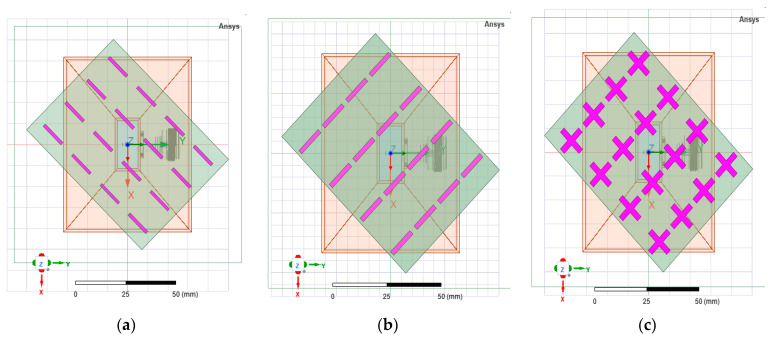
Front view of the FSS structures for three radiator shapes, left tilted: “\” (**a**), “/” (**b**) and “+” (**c**).

The distances between successive layers were calculated in a similar manner to when designing a Yagi-Uda array, using a dedicated online calculator. The resulting distances between the five layers of the FSS structure are given in Table 2. Based on the analysis presented in Section 3.2.1, the FSS structure consists of 4 × 4 radiators. Three geometries of FSS structures were investigated (Figure 11) based on the three types of unit cell analyzed in Section 2.2 by varying the width of the radiators (1.5 mm, 2 mm, 2.5 mm and 3 mm), but also the number of layers of FSS structures placed in front of the aperture of the pyramidal horn.

The simulations for the horn antenna with the structures presented in Figure 11 were performed by rotating the FSS structures at ±45°. The best figures of merit were obtained for the versions 1, 2, 5 and 8 as displayed in Table 3. The criterion for choosing the best version was based on a tradeoff between axial ratio and the difference between the gain figures for cross-polarization and co-polarization, respectively.

**Figure 12:**
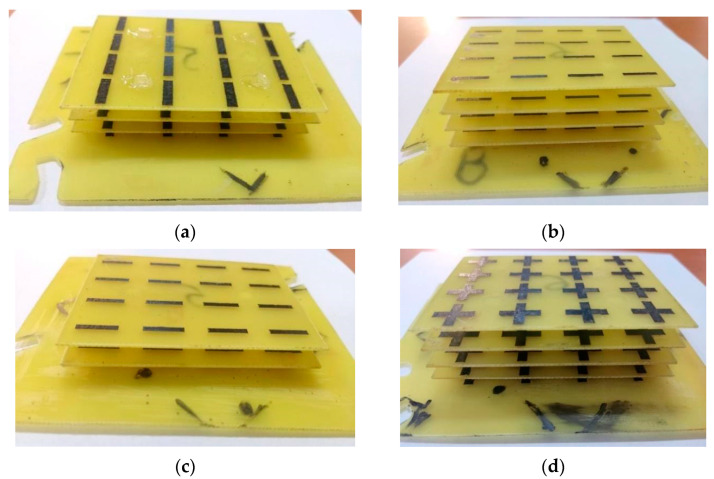
Fabricated FSS structures: type 5 (**a**), type 1 (**b**), type 2 (**c**) and type 8 (**d**).

Figure 12 shows the physical FSS structures for each selected version. To make it easier to follow, we will designate them from now on as denoted in Figure 12: type 5 (a), type 1 (b), type 2 (c), and type 8 (d). The distance between the physical FSS layers was kept as in Table 2 by using 3D-printed carbon fiber spacers.

### 3.3. Simulation and Measurement Results

The measurements were carried out inside an anechoic chamber. The probe antenna and the antenna under test were connected to a vector network analyzer after compensating the cable effects by performing a calibration. The probe antenna was a broadband ridged horn operating from 700 MHz to 18 GHz, with a gain varying between 2 and 17 dBi. The distance between the two antennas was set to 2400 mm.

The polarization pattern of the measured antenna was drawn by rotating the probe antenna in the E-plane with an angular step of 45°, and the radiation pattern by rotating the antenna under test in the H-plane, respectively.

**Figure 13:**
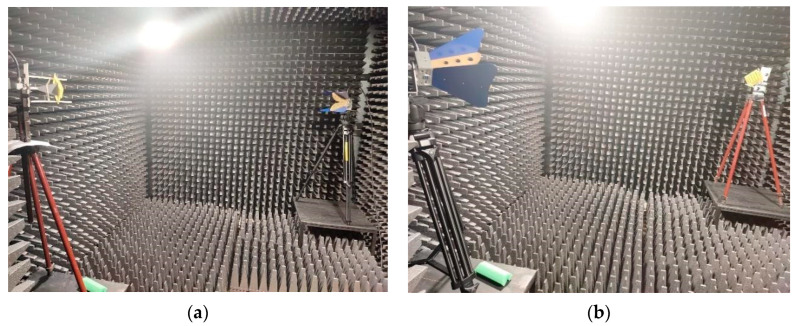
Measurement setup in the anechoic chamber for radiation pattern (**a**) and polarization pattern (**b**).

**Figure 14:**
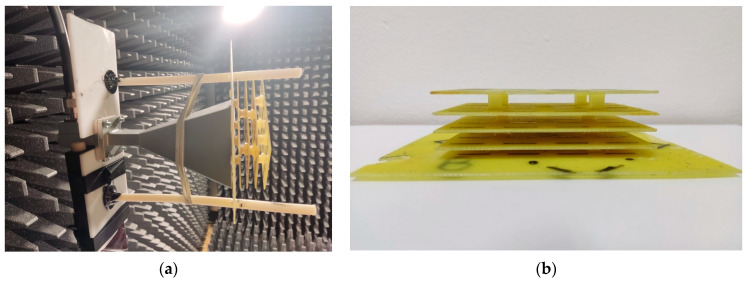
Radiating system consisting of a conventional pyramidal horn antenna with a multi-layer FSS structure (**a**) and FSS structure with “|”-type radiators—side view (**b**).

The measurement setup for antenna gain is presented in Figure 13a, and for antenna polarization in Figure 13b, respectively. Figure 14a shows the radiating system under test consisting of the general purpose, pyramidal horn antenna and different types of FSS structure. The side view of the FSS structure with “|” type radiators can be seen in Figure 14b.

#### 3.3.1. Input Reflection Coefficient

**Figure 15:**
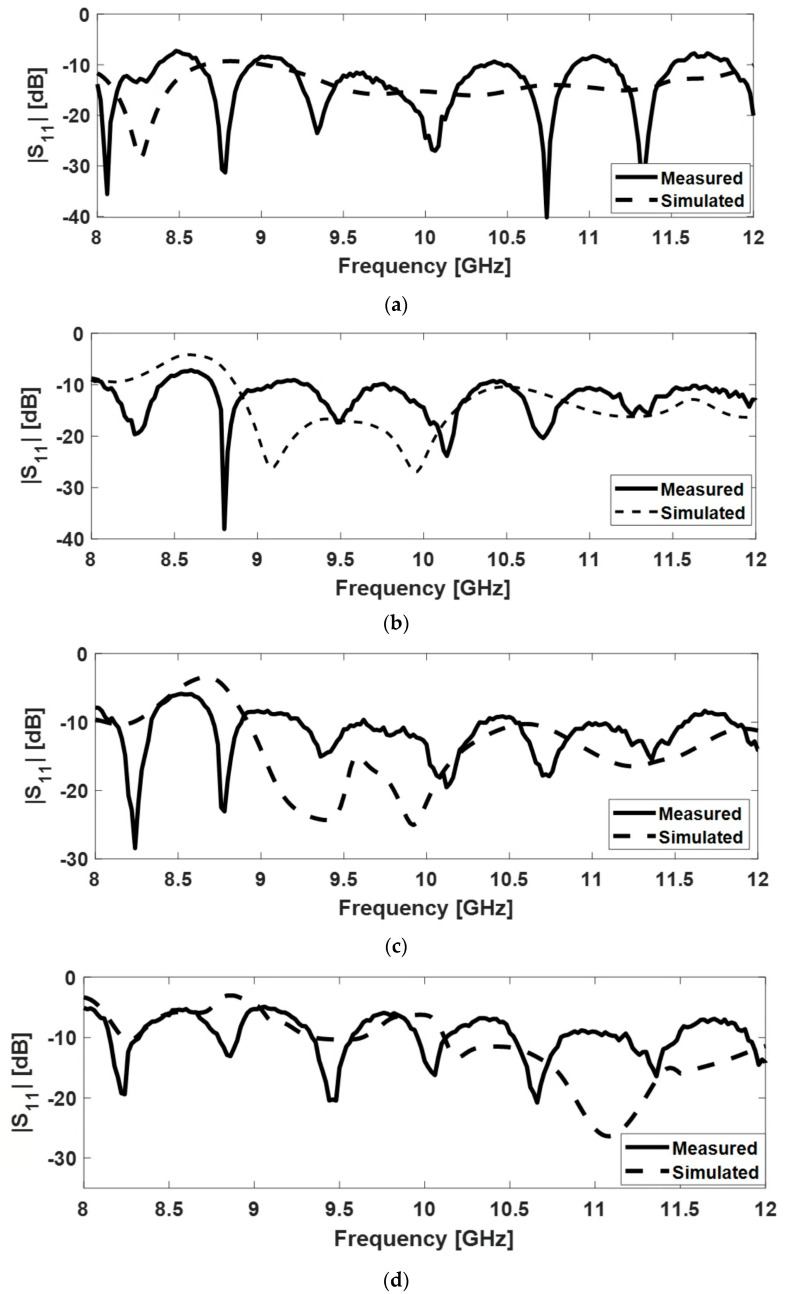
Magnitude of the input reflection coefficient for FSS-horn structures: versions (**a**–**d**).

The variation of $\left|S_{11}\right|$ with the frequency for the four FSS-horn structures is shown in Figure 15. Measurement and simulation results are included in the same graph for comparison. The $\left|S_{11}\right|$ for all four versions is below −11 dB over the entire frequency band in the simulations, and below −5 dB in the measurements.

#### 3.3.2. Radiation Patterns

**Figure 16:**
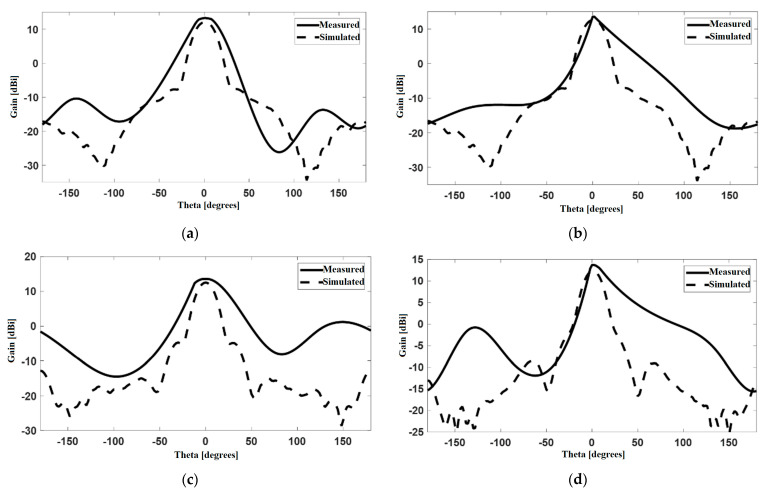
H-plane radiation pattern of the FSS-horn structure at 12 GHz: versions (**a**–**d**).

The radiating system under test was placed such that the horn exciting the FSS structure shares the same polarization with the probe antenna. Figure 16 shows a comparison between the radiation pattern of our radiating system resulting both from simulation and measurements. The results show a good agreement in the main direction of radiation, i.e., along the *z*-axis (θ=0°). The difference between measurements and simulations in Figure 16b,d is mainly due to the influence of the dielectric plate placed behind the horn antenna in order to hold the FSS structure; that dielectric plate actually reflects the field back scattered by the FSS layers.

**Figure 17:**
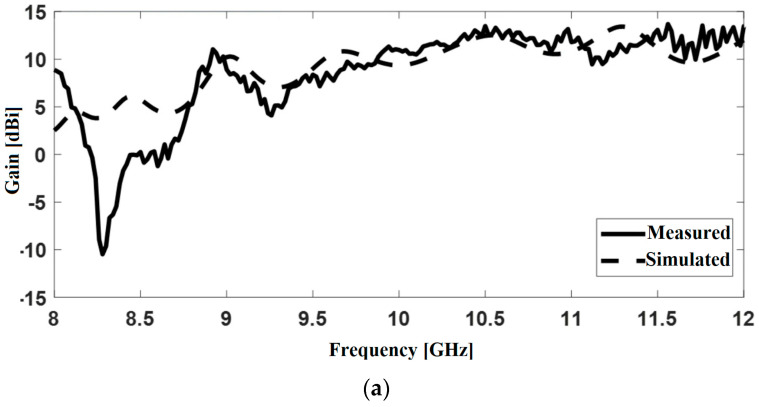

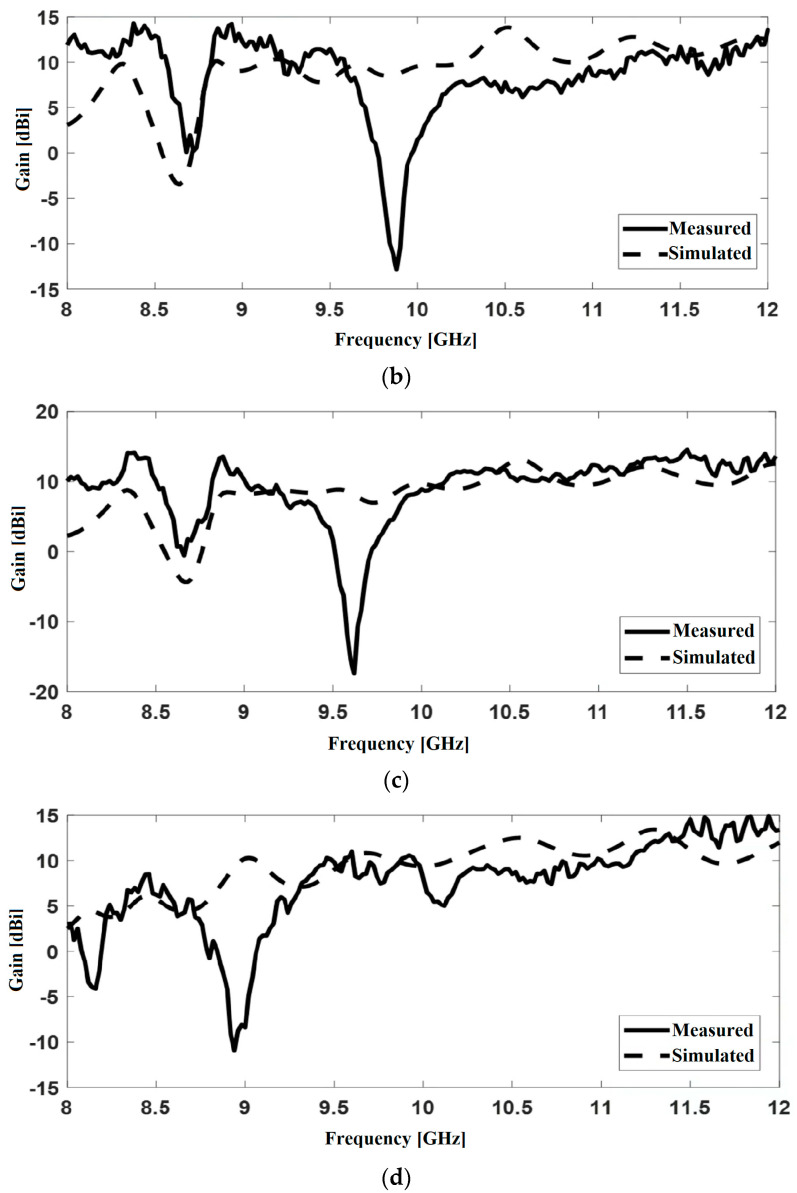
Simulated and measured gain in the main direction of radiation: versions (**a**–**d**).

Figure 17 shows the H-plane gain measured in the main direction of radiation as a function of frequency, and the simulated gain. The root mean square error on the gain figure is: 0.11 dB for version (a), 0.15 dB for version (b), 0.05 dB for version (c), and 0.008 dB for version (d). A good agreement between measured and simulated results can be noted.

#### 3.3.3. Polarization

The polarization of the antenna under test can be determined by rotating the linearly polarized probe antenna in the E-plane.

**Figure 18:**
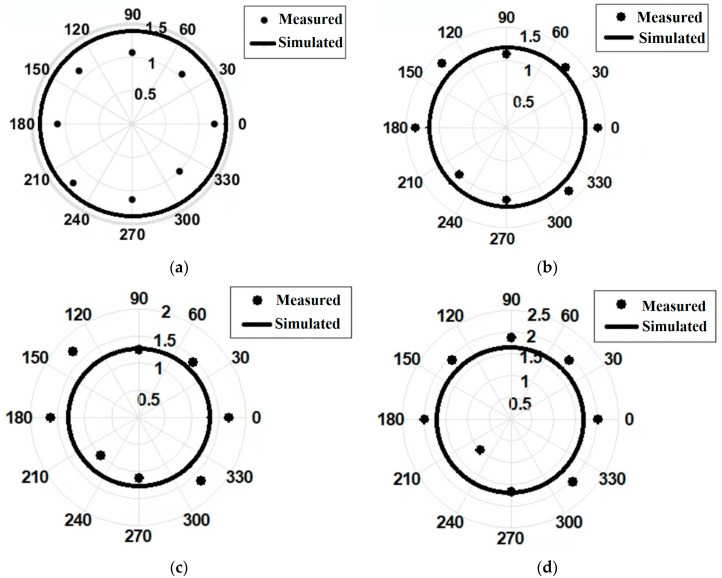
E-plane axial ratio as a function of azimuth angle (φ) at 12 GHz: versions (**a**–**d**).

**Figure 19:**
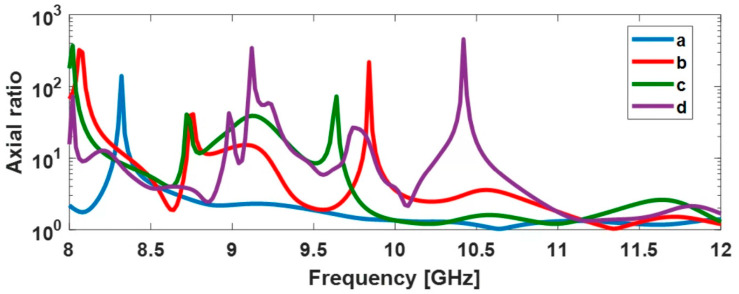
Simulated axial ratio as a function of frequency for θ=0° and Φ=0°.

Figure 18 plots the magnitude of the axial ratio as a function of the rotation angle of the probe antenna (°) and shows that our horn-FSS antenna provides a circular polarization, as expected. The root mean square error for each structure is: 0.26 for version (a), 0.16 for version (b), 0.58 for version (c), and 0.28 for version (d). Figure 19 exhibits an axial ratio close to 1 and quasi-constant between 11 and 12 GHz for version (a), which means that the objective of changing the polarization of a conventional horn antenna by adding successive layers of frequency-selective surfaces has been achieved over the frequency range assigned to the intended applications. A full comparison between simulated and measured figures of merit is presented in Table 4.

## 4. Conclusions

In this paper, a simple and low-cost method for changing the polarization of a conventional pyramidal horn antenna was presented. Our technique consists of adding several layers of frequency-selective surfaces in front of the aperture of the pyramidal horn. The FSS structure was rotated at 45° with respect to the aperture vertical axis in order to transform a linearly polarized electric field into two components of the same magnitude and phase shifted by 90°. The position of the FSS structures relative to the horn aperture was chosen by analogy with the Yagi-Uda arrays. The size of the skewed, FSS layers consisting of λ/2 passive dipoles should be the same as the horn aperture size; simulations with larger surfaces showed no further improvement in terms of relevant figures of merit. By comparing the results for several types of FSS structures, it comes out that the version denoted by (a), and consisting of four layers of four-by-four linear half-wave dipoles provides the best figures of merit: overall gain of 12 dBi, axial ratio close to 1 in the E-plane and quasi-constant between 11 and 12 GHz, together with a good impedance matching over the frequency range of interest. The overall gain of our radiating system increased by 3 dB compared to the original pyramidal horn antenna.

The major advantage of the proposed FSS-horn radiating system is the manufacturing cost, since a conventional, general-purpose horn antenna can be easily converted into a CP antenna with FSS printed on a substrate with a single metal layer.

Future research will focus on implementing a set of two FSS-horn antennas in a system for determining the direction of arrival of a radio wave.

## Figures and Tables

**Table 1:** Figures of merit for the radiating system consisting of a horn antenna and a single-layer FSS structure with *N* × *N* radiators.

Number of Elements of the FSS Structure, *N* × *N*	Overall Gain, *G* [dB]	Gain Difference between Types Circular Polarization, *G_RHCP_* − *G_LHCP_* [dB]	Axial Ratio
2 × 2	5.2	0.3	58
3 × 3	11.34	1.74	10
4 × 4	13.02	2.3	7
5 × 5	12.7	2.8	6
6 × 6	8.64	2.36	7.38
7 × 7	12	2.5	6.86

*G_RHCP_*—right hand circular polarization gain; *G_LHCP_*—left hand circular polarization gain.

**Table 2:** Finding the position of the FSS layers with respect to the horn aperture.

	Vibrator (Horn Aperture)	Director 1	Director 2	Director 3	Director 4	Director 5
Position [mm]	0	2	6	12	18	25

**Table 3:** Figures of merit of the antenna system for the six types of FSS structure.

Ref. #	FSS Structure Type (Figure 11)	Number of Layers	Radiator Width [mm]	Overall Gain, *G* [dBi]	Gain Difference between Types of Circular Polarization, *G_RHCP_* − *G_LHCP_* [dB]	Axial Ratio	Gain Difference between Types of Linear Polarization, *G_co_* – *G_cross_* [dB]	Dominant Circular Polarization
1	“\”, left tilted	5	1.5	12.5	21.32	1.19	1.36	RHCP
		1	1.5	10.6	4.1	4.24	10.27	RHCP
2		3	2	12.6	18	1.28	0.9	RHCP
		1	2	12	4	3.66	10	RHCP
3	“\”, right tilted	5	1.5	12,5	21	1,19	1.43	LHCP
		1	1.5	12.2	4	4.31	11.3	LHCP
4	“/”, left tilted	3	2.5	13.2	13.08	1.56	3.92	LHCP
		1	2.5	11.2	3.5	5.15	12.8	LHCP
5		4	3	12	15.77	1.38	2.14	LHCP
		1	3	10.9	4	4.47	12.5	LHCP
6	“/”, right tilted	4	3	11.4	16	1.38	1.8	RHCP
		1	3	12.3	4	4.46	12	RHCP
7		4	2.5	12.4	11.13	1.76	2.18	RHCP
		1	2.5	8.3	3	5.19	14	RHCP
8	“+”, left tilted	5	3	12.2	12	1.67	2.42	RHCP
		1	3	10.6	3	5.9	12.3	RHCP
9	“+”, right tilted	5	2.5	12.5	10.56	1.84	4.37	LHCP
		1	2.5	6.1	2.5	7	15	LHCP
10		4	3	11.73	11.4	1.73	4.77	LHCP
		1	3	1.85	2	5.69	11	LHCP

*G_co_*—co-polarization gain; *G_cross_*—cross-polarization gain.

**Table 4:** Simulated and measured results for the four radiating systems with multi-layer FSS structures.

Ref. #	FSS Structure	Number of layers	Radiator Width [mm]	Overall Gain, *G* * [dBi]	Gain Difference between Types of Circular Polarization, *G_RHCP_* − *G_LHCP_* [dB]	Axial Ratio *	Gain Difference between Types of Linear Polarization, *G_co_* – *G_cross_* * [dB]	Dominant Circular Polarization
(a)	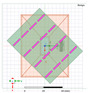	4	3	12/13.28	15.77	1.38/ 1.22	2.14/ 1.22	LHCP
(b)	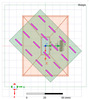	5	1.5	12.5/13.53	21.32	1.19/ 1.4	1.36/ 2	RHCP
(c)		3	2	12.6/13.62	18	1.28/ 2.5	0.9/ 3.5	RHCP
(d)	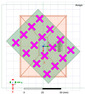	5	3	12.2/ 13.72	12	1.67/ 2	2.42/ 0.6	RHCP

* Simulated/measured values.

## Data Availability

The data that support the findings of this study may be available from the corresponding author upon reasonable request.
